# Retinal organoid–based evaluation of icariin-loaded extracellular vesicles for hypoxia-induced retinal injury via mitochondrial modulation

**DOI:** 10.3389/fcell.2026.1891491

**Published:** 2026-09-28

**Authors:** Mira Park, Hyeoun Ji Kim, Yoon-Ha Go, Hey Jin Lee, Young Il Yang, Dong Hyuck Bae, Jong man Yoo, Helen Lew

**Affiliations:** 1 Department of Ophthalmology, CHA Bundang Medical Center, CHA University, Seongnam, Gyeonggi-do, Republic of Korea; 2 Red-Green Bioconvergence Institute, ORGANOIDSCIENCES CO., LTD., Iksan, Jeonbuk-do, Republic of Korea; 3 ORGANOIDSCIENCES CO., LTD., Cheongju, Jeonbuk-do, Republic of Korea; 4 CHA R&D Institute, CHA University, Seongnam, Gyeonggi-do, Republic of Korea; 5 Paik Institute for Clinical Research, Inje University College of Medicine, Busan, Republic of Korea; 6 Integrated Medicine Major (IM), Biomedical & Health Service Convergence Division, Graduate School of JABA, WonKwang University, Iksan, Republic of Korea; 7 VOS DISCOVERY CO., LTD., Ho Chi Minh, Vietnam

**Keywords:** extracellular vesicles (EVs), hypoxia, icariin, mitochondria, retinal organoid

## Abstract

**Background:**

Hypoxic injury is a critical pathological factor in various retinal degenerative diseases, leading to mitochondrial dysfunction and irreversible loss of retinal neurons. This study investigated the neuroprotective potential of Icariin-loaded extracellular vesicles (EV_ICA) using both R28 retinal progenitor cells and human embryonic stem cell (hESC)-derived retinal organoids.

**Methods:**

EV_ICA were isolated and characterized by morphology, EV marker expression, and zeta potential analysis. A CoCl_2_-induced hypoxic injury model was established in R28 cells and hESC-derived retinal organoids to evaluate the therapeutic effects of EV_ICA. Gene and protein expression analyses, bulk RNA sequencing (RNA-seq), gene set enrichment analysis (GSEA), mitochondrial ROS measurements, and intracellular ATP assays were performed to assess neuroprotective efficacy and mitochondrial function.

**Results:**

EV_ICA exhibited typical spherical morphology, expressed standard EV markers, and showed a shift toward a less negative zeta potential compared to Naïve EV. In hypoxia-injured retinal models, EV_ICA significantly restored the expression of key retinal and neuroprotective markers, including Syntaxin12, NeuN, Vegf, Rbpms, Brn-3a, and Bdnf, while suppressing Hif-1α accumulation. Transcriptomic and GSEA analyses revealed that EV_ICA reactivated biological pathways associated with phototransduction, visual perception, and retinal development that were impaired under hypoxic conditions. Furthermore, EV_ICA improved mitochondrial homeostasis by up-regulating mitochondrial-related genes, reducing mitochondrial ROS accumulation, and recovering intracellular ATP production.

**Conclusion:**

These findings demonstrate that EV_ICA exerts neuroprotective effects by preserving mitochondrial quality control and restoring functional retinal gene programs under hypoxic stress. EV_ICA may therefore serve as a promising therapeutic candidate for hypoxia-related retinal degeneration and provide potential applications for cell-free regenerative medicine.

## 1 Introduction

Optic nerve injury (ONI), encompassing traumatic optic neuropathy and glaucomatous optic neuropathy, induces irreversible retinal ganglion cell (RGC) death via oxidative stress, neuroinflammation, apoptosis, and impaired CNS regeneration (Mead and Tomarev, 2017). Despite corticosteroids offering limited neuroprotection, effective regenerative therapies remain urgently needed. Our previous studies established foundational ONI models and therapeutic scaffolds, demonstrating significant RGC survival improvements in rat optic nerve crush assays (Park et al., 2022) and scaffold-mediated axon regeneration (Shin et al., 2023).

Despite advances in neuroprotection and regeneration research, clinical translation has been limited by the blood-retina barrier, chronic inflammation, and disrupted mitochondrial homeostasis in the optic nerve microenvironment. Mesenchymal stem cell (MSC) therapy has emerged as a promising approach due to its paracrine effects, but challenges including tumorigenicity, immunogenicity, and poor engraftment persist (Park et al., 2022). EVs, nanoscale lipid-bilayer particles secreted by MSCs, offer superior alternatives by transferring bioactive cargos—miRNAs, proteins, and lipids—while eliminating cellular risks. EVs exhibit enhanced biocompatibility, blood-retina barrier permeability, and scalability for clinical use, promoting RGC survival and axonal regeneration (Koh et al., 2020; Park et al., 2022). Mesenchymal stem cell (MSC)-derived EVs mediate neuroprotection through miRNAs (e.g., miR-182), growth factors (BDNF), and signaling modulation (PTEN/mTOR, PI3K/Akt), enhancing RGC survival and axon regrowth in ONI models (Koh et al., 2020). Cardiac MSC (CMSC)-EVs exhibit superior anti-inflammatory and angiogenic profiles via enriched miR-19a and VEGF cargos (Xiao et al., 2016). In addition to their pro-angiogenic effects, EVs derived from human myocardium-derived adult stem cells (MyoCSCs) have shown strong regenerative potential. Previous studies demonstrated that MyoCSC-derived EVs promote tissue repair by enhancing cell proliferation and angiogenesis, leading to improved wound healing (Kang et al., 2020). In addition, stem cell-derived EVs have been reported to protect retinal ganglion cells and reduce oxidative stress and mitochondrial damage in optic nerve injury models (Park et al., 2022). Since oxidative stress, mitochondrial dysfunction, and neuronal apoptosis are common features of both optic nerve injury and retinal ischemia, these findings support the potential use of MyoCSC-derived EVs for retinal neuroprotection.

In ONI models, EVs mitigate oxidative stress, apoptosis, and demyelination more effectively than MSCs, with improved mitochondrial transfer restoring bioenergetics in injured neurons (Park et al., 2022). Naive EVs, however, are limited by heterogeneous endogenous cargo and suboptimal delivery efficiency at injury sites. Engineered (loaded) EVs, enriched with neuroprotective agents like BDNF or mitochondrial-targeted antioxidants, overcome these limitations by enabling precise, high-dose delivery. This customization enhances anti-inflammatory signaling, synaptic remodeling, and long-term functional recovery beyond naive EVs, positioning loaded EVs as optimized therapeutics for ONI (Koh et al., 2020; Park et al., 2022).

Icariin, an Epimedium flavonoid, exerts potent antioxidant effects (Nrf2/HO-1 upregulation), attenuates inflammation (NF-κB inhibition), and prevents apoptosis (JNK/p38 suppression) in ischemic ONI models, preserving RGC function (Pan et al., 2014). Icariin (ICA), a flavonoid glycoside from Epimedium species, was selected as the optimal cargo for EV loading due to its multifaceted neuroprotective profile in nerve injury models (Wang et al., 2022). ICA robustly activates PI3K/Akt and ERK pathways to inhibit apoptosis, suppress neuroinflammation via NF-κB downregulation, and preserve mitochondrial integrity by modulating Bcl-2/Bax balance and ROS scavenging (Zhang et al., 2015; Zhang et al., 2019). In retinal and optic nerve injury studies, ICA promotes RGC survival, axonal regrowth, and visual function recovery at low micromolar doses, outperforming other flavonoids in potency and bioavailability (Nakayama et al., 2011; Desai et al., 2022). Loading ICA into EVs addresses its key limitations: poor aqueous solubility, rapid hepatic metabolism, and suboptimal CNS penetration (Zhang et al., 2022). EV encapsulation enhances ICA stability, prolongs circulation (half-life >24 h), and facilitates targeted delivery across the blood-retina barrier via CD63-mediated endocytosis in neurons and glia (Men et al., 2019; Pollalis et al., 2022). Synergistically, EV_ICA combines ICA’s bioactivity with EV-mediated mitochondrial transfer and miRNA modulation, yielding superior outcomes in ONI models compared to free ICA or naive EVs (Wang et al., 2022; Tang et al., 2024). This rationale positions EV_ICA as a clinically viable, precision-engineered therapy for ONI neuroprotection and regeneration.

*In vitro* validation using R28 retinal cells (hypoxia/CoCl_2_ injury models) demonstrates EV-mediated RGC protection, neurite outgrowth, and miRNA modulation (e.g., miR-374b-5p), while retinal organoids recapitulate 3D architecture for assessing tissue-level RGC survival and axonal integration (Capowski et al., 2019). This study evaluates Icariin-loaded EVs across R28 cells and retinal organoids as hypoxic injury models to elucidate synergistic mechanisms and therapeutic potential for clinical translation.

## 2 Methods

### 2.1 Preparation of EVs

EVs from MyoCSCs (Kim et al., 2015; Kang et al., 2020) were obtained from Prof. Young Il Yang (Inje University College of Medicine, Busan, Republic of Korea). The EVs were manufactured by Matica Biolabs at the GMP facility of Bundang CHA Hospital (Seongnam, Republic of Korea).

### 2.2 Preparation of EV_ICA

EV_ICA was prepared according to a previously reported protocol (Dong et al., 2021). Briefly, Icariin (ICA) was dissolved to prepare a 1 mg/mL stock solution. For drug loading, ICA was mixed with EVs at a 1:9 ratio and incubated for 24 h at room temperature to allow passive loading by co-incubation. Following incubation, the mixture was centrifuged at 1,000 × g for 10 min to remove free ICA aggregates. The supernatant was subsequently ultracentrifuged at 135,000 × g for 2 h to pellet EV_ICA. The EV_ICA pellet was resuspended in sterile phosphate-buffered saline (PBS), passed through a 0.22-μm syringe filter, and stored at −80 °C until use. The concentration of EV_ICA was determined using the bicinchoninic acid (BCA) protein assay, and all experimental doses were normalized based on EV protein concentration.

### 2.3 Characterization of EVs

The size and zetapotential of particles were determined using a MONO ZetaView® (PMX-120; Particle Metrix, Meerbusch, Germany) in 488 nm scatter mode. The EVs samples were diluted to 10^7^–10^8^ particles/mL using filtered phosphate-buffered saline (PBS) (HyClone Laboratories, Logan, UT, USA) or distilled water. For all samples, the detailed parameters for accurate analysis were tuned with a sensitivity 75, shutter of 100, minimum trace length of 15, and cell temperature of 25 °C.

### 2.4 Cell culture and EVs treatment

Immortalized R28 retinal precursor cells were cultured and treated with EVs as described previously (Park et al., 2022). A hypoxic environment was produced in cells by treatment with cobalt chloride (CoCl_2_; Sigma-Aldrich, St. Louis, MO, USA). Aliquots of 2 × 10^5^ R^28^ cells were seeded onto glass coverslips and treated with 200 µM CoCl_2_ for 6 h. The hypoxia-damaged R28 cells were then treated with EVs 170–180 nm in diameter at a concentration of 24 μg/mL. After 30 h, the cells were harvested and prepared for analysis.

### 2.5 Immunoblot analysis

Total proteins from cells or tissues were extracted using PRO-PREP buffer (iNtRON Biotechnology, Gyeonggi-do, Republic of Korea). Protein concentration was determined by bicinchoninic acid assay (BCA) (Thermo Fisher Scientific). Target proteins were separated by SDS-PAGE and transferred onto PVDF membranes (GE Healthcare, Chicago, IL, USA), which were then incubated overnight at 4 °C with primary antibodies (Supplementary Table S1). After a series of washes, the membranes were incubated overnight at 4 °C with horseradish peroxidase-conjugated anti-rabbit or mouse secondary antibody at 1:10,000 dilution (GeneTex, Irvine, CA, USA). Target bands were detected on an ImageQuant™ LAS 4000 (GE Healthcare).

### 2.6 Maintenance of human embryonic stem cells for organoids

Human embryonic stem cells (hESC; H9(WA09) cell line) were obtained from the WiCell Stem Cell Bank. The WA09 cell line was cultured in mTeSR1 medium (Stem Cell Technologies, Vancouver, BC, Canada) in 60-mm culture dishes (Wuxi Nest Biotechnology, Wuxi, Jiangsu, 214,112, China), which were coated with hESC-qualified Matrigel (Corning, Tewksbury, MA 01876, USA), at 37 °C with 5% CO_2_ overnight. hiPSCs are maintained with fresh media daily. Passaging was conducted every 4–5 days by treating the hESC colonies with Accutase (Stem Cell Technologies) at 37 °C for 5 min when 70%–80% confluency. Following the detachment of hESCs as single cells, they were resuspended in mTeSR1 supplemented with 20 µM Y-27632 (Tocris, Bristol, City of Bristol, United Kingdom), pelleted by centrifugation at 1,350 rpm for 10 min at room temperature, and subsequently passaged at a ratio 1:10 to 1:50. After passaging, the remaining cells are stored at −80 °C in a stock solution containing 90% KnockOut Serum Replacement (KOSR; Gibco, Waltham, MA, USA) and 10% dimethyl sulfoxide (DMSO; Duchefa Biochemie, Haarlem, North Holland, Netherlands).

### 2.7 Generation of hESC-derived retinal organoids

The formation of embryonic bodies (EBs) involved the dissociation of human embryonic stem cells (hESCs) into individual cells using Accutase. The cells were then cultured in 96-well V-bottom plates (PrimeSurface, Tokyo, Japan) at a density of 1.2 × 10^4^ cells per well. The culture was maintained in Retinal Embryonic Body media (RB media) supplemented with 20 µM Y-27632 at 37° in a 5% CO2 environment. The RB media is supplemented with 45% IMDM medium (Gibco, Waltham, MA, USA), 45% F12 medium (Invitrogen, Waltham, MA, USA), 10% Knockout Serum Replacement (Gibco), 1X Glutamax (Gibco), 1X Chemically Defined Lipid Concentrate (Invitrogen), and 450uM monothioglycerol (Sigma-Aldrich). Then, on day 3, the culture medium was changed to full RB media with 20 µM Y-27632. After 6 days, a complete medium change to RB media supplemented with 55 ng/mL BMP4 (Gibco) was performed, with subsequent BMP4 dilutions conducted through half changes to RE media on days 9, 12, and 15 (Yamasaki et al., 2022).

On day 18 of retinal organoid differentiation, EBs were transferred from 96 well V-bottom plates to 60 mm petri dishes at a density of 12–24 organoids/dish. For RPE induction, the cultures were maintained until days 22–24 for optimal induction in a shaker incubator at 37 °C with 5% CO2. The retinal pigment epithelium media (RPE media) was supplemented with DMEM/F12-Glutamax medium (Gibco), 1X N-2 (Gibco), 3 µM CHIR 99021 (PeproTech, Cranbury, NJ, USA), and 5 µM SU5402 (Sigma-Aldrich).

Beginning on day 24 of differentiation, the cultures were transitioned to Retinal Epithelium Media (RE media) and maintained under conditions that included media changes every 2–3 days. The RE media was supplemented with DMEM/F12-Glatmax medium, 1X N-2, 10% Fetal Bovine Serum (FBS), 0.5 µM Retinoic Acid (Sigma-Aldrich), 0.1 mM Taurine (Sigma-Aldrich), and 0.25 μg/mL Amphotericin B (Invitrogen). Following day 24, the cultures were maintained at 37 °C Celsius under 5% CO2 conditions in a shaking incubator. To ensure the reproducibility of the organoid model, the differentiation efficiency was determined individually for five independent batches: Batch 1 (65%), Batch 2 (62%), Batch 3 (58%), Batch 4 (50%), and Batch 5 (52%), yielding a mean ± SD of 55% ± 3%. The low batch-to-batch variance demonstrates the robustness and reproducibility of the organoid differentiation protocol.

### 2.8 Retinal organoid hypoxia-induced modeling

Hypoxia modeling was performed by incorporating cobalt chloride 0.1 M solution (CoCl_2_; Sigma-Aldrich) at varying concentrations (0, 600, 1,200 µM) into 2 mL of RE media in a 6-well non-treat plate. The plate was then incubated for designated assay periods of 24 h and 48 h. The CoCl_2_ treatment was conducted over a 12-h period, during which the exosome samples were introduced at concentrations of 0, 100, and 200 µg. The mixture was then incubated for an additional 24 h. For each experimental group, at least 3–5 organoids were utilized, and to ensure reproducibility, all organoids were derived from the same cell line and processed across five independent differentiation batches. To minimize selection bias, all organoids used in the experiment were randomly assigned to each group and undifferentiated organoids were strictly excluded from the study. Organoids were selected based on a semi-quantitative scoring procedure. Immunofluorescence images of each organoid section were captured under identical conditions using a Nikon Eclipse Ti2 microscope (Nikon, Tokyo, Japan). Slides were coded to blind the evaluators to the treatment groups. Two independent evaluators quantified the proportion of the laminar circumference displaying continuous and correctly localized expression of four key markers: PAX6, BRN3a, RBPMS, and Recoverin. Only organoids with a score of ≥70% from both evaluators were included. A third blinded reviewer subsequently resolved discrepancies in scoring.

### 2.9 H&E and immunoflurorence for histology

Retinal organoids were fixed with 4% paraformaldehyde (Bio-solution, Suwon-si, South Korea). Paraffin blocks were sectioned to a thickness of 4 μm, paraffin slide were deparaffinized. For histological analysis, the sections were stained with hematoxylin and eosin (Hematoxylin; Cambridge, United Kingdom and Eosin; Cambridge, United Kingdom). For immunofluorescence, antigen retrieval was performed using sodium citrate (Sigma, St. Louis, MO, USA) buffer containing Tween 20, pH 6.0. The primary antibody (Supplementary Table S1) was diluted in 1% BSA (Bovine Serum Albumin; Sigma-Aldrich) and incubated overnight at 4°. Shade-light blocking-throughout all subsequent stages. The secondary antibody was treated, and Hoechst stain was utilized as a final step. The samples were stored at 4 °C prior to imaging. To evaluate the quality of the differentiated retinal organoids, histological stratification was semi-quantitatively assessed using paraffin-sectioned samples. Proper retinal stratification was defined by the presence of distinct laminar structures and the correct spatial organization of layer-specific retinal cell types. Specifically, organoids were qualified and included in downstream experimental groups only when more than 70% of the continuous laminar structure exhibited a clear and organized co-expression of all four key retinal markers: PAX6, BRN3a, RBPMS, Recoverin, within their respective layers. Conversely, any organoids showing fragmented marker expressions, disorganized cellular alignment, or an absence of these target layers (covering less than 30% of the cross-sectional area) were quantified as poorly stratified or undifferentiated and were strictly excluded from the study.

### 2.10 TUNEL assay

Apoptotic cell death in retinal organoids was assessed using the *In Situ* Cell Death Detection Kit, FITC (Roche, Mannheim, Germany) according to the manufacturer’s instructions with minor modifications. Following experimental treatments, retinal organoids were fixed in 4% paraformaldehyde overnight at 4 °C, dehydrated through a graded ethanol series, cleared in xylene, and embedded in paraffin. Paraffin blocks were sectioned at 5 μm thickness and mounted onto poly-L-lysine–coated glass slides. For TUNEL staining, sections were deparaffinized, rehydrated, and treated for antigen retrieval. The TUNEL reaction mixture containing terminal deoxynucleotidyl transferase (TdT) enzyme and FITC-labeled nucleotides were applied to each section, followed by incubation at 37 °C for 60 min in a humidified dark chamber. Negative controls were processed without TdT enzyme, whereas positive controls were pretreated with DNase I (1 U/μL) before the TUNEL reaction. After washing with PBS, nuclei were counterstained with DAPI and mounted using an anti-fade mounting medium. Fluorescence images were obtained using a slide scanner (Axio Scan. Z1; Carl Zeiss) under identical imaging conditions for all groups. TUNEL-positive cells were quantified across the entire retinal layer in three to five randomly selected non-overlapping fields per section. The percentage of TUNEL-positive cells relative to total DAPI-positive nuclei was analyzed using ZEN Blue software (version 3.1; Carl Zeiss Microscopy GmbH, Jena, Germany). Data were obtained from at least three independent biological replicates per group and expressed as mean ± standard deviation (SD).

### 2.11 Bulk RNA seqencing

Retinal organoids were treated with 600 μM CoCl_2_ to induce hypoxic injury. Following hypoxic induction, organoids were treated with 100 μg/mL of naïve EV or EV_ICA for 24 h. Total RNA was extracted from retinal organoids and subjected to bulk RNA sequencing (RNA-seq).

RNA libraries were prepared for 151-bp paired-end sequencing using the TruSeq Stranded Total RNA Kit (Illumina, CA, USA). Ribosomal RNA was removed using biotinylated, target-specific oligonucleotides combined with Ribo-Zero rRNA Removal Beads (Illumina) from 1 μg of total RNA. The remaining RNA was purified and fragmented. Fragmented RNAs were reverse-transcribed into first-strand cDNA using random hexamer primers, followed by second-strand cDNA synthesis. After end repair, A-tailing, adapter ligation, and PCR amplification, library quality was evaluated using the TapeStation 4,200 instrument and D1000 ScreenTape System (Agilent Technologies, CA, USA). Libraries were quantified using the KAPA Library Quantification Kit (Kapa Biosystems, MA, USA) according to the manufacturer’s instructions. Sequencing was performed as 151-bp paired-end reads (2 × 151 bp) using the Illumina NovaSeq 6,000 platform (Illumina, CA, USA).

Raw sequencing reads were filtered to remove low-quality reads according to the following criteria: reads containing >10% ambiguous bases (N), >40% bases with quality scores <20, or an average quality score <20. Filtering was performed using in-house scripts. Filtered reads were aligned to the reference genome using STAR v2.4.0b (Dobin et al., 2013). Gene expression levels were quantified using Cufflinks v2.1.1 (Trapnell et al., 2010) with the species-specific gene annotation database. Non-coding gene regions were excluded using the–mask option, and multi-read-correction and frag-bias-correct options were applied to improve expression accuracy. Gene-level read counts were generated using HTSeq-count v0.6.1p1 (Anders et al., 2015). Differentially expressed genes (DEGs) were identified using the TCC R package (Sun et al., 2013), which applies iterative DEGES/edgeR normalization. P-values were adjusted using the Benjamini–Hochberg method (Benjamini and Hochberg, 1995), and genes with an adjusted q-value <0.05 were considered differentially expressed.

### 2.12 Transcriptome data analysis

#### 2.12.1 Gene Ontology and pathway enrichment analysis

Functional enrichment analyses, including Gene Ontology (GO) and Kyoto Encyclopedia of Genes and Genomes (KEGG) pathways, were implemented using the clusterProfiler R package (v4.16.0). The *Homo sapiens* annotation database (org.Hs.eg.db) was utilized for gene mapping. GO enrichment was assessed across the Biological Process (BP), Cellular Component (CC), and Molecular Function (MF) domains. For each domain, the top seven terms ranked by q-value were selected and visualized as bar histograms. Gene-to-term relationships were illustrated as chord diagrams using the circlize R package. For each comparison, up to 30 representative genes were selected based on log2 fold change per term, and chord connections were color-coded by log2 fold change (blue: downregulated, red: upregulated). KEGG pathways were categorized according to the KEGG BRITE hierarchy and visualized as bar histograms representing the number of unique genes per subcategory, grouped by top-level functional categories. Additionally, KEGG dot plots depicting GeneRatio and statistical significance were generated using ggplot2.

#### 2.12.2 Mitochondria-associated gene expression and GSEA analysis

Expression profiles of 22 mitochondria-associated genes were extracted from TPM-normalized RNA-seq data obtained from the Control, CoCl_2_, CoCl_2_+EV, and CoCl_2_+EV_ICA groups. Gene expression values were Z-score normalized across samples, and hierarchical clustering and heatmap visualization were performed using the ComplexHeatmap R package (v2.24.1).

Gene Set Enrichment Analysis (GSEA) was performed using the gseGO and gseKEGG functions in the clusterProfiler package. Protein-coding genes were ranked according to log_2_ fold change for the following comparisons: (1) CoCl_2_ vs. Control, (2) CoCl_2_+EV vs. CoCl_2_, (3) CoCl_2_+EV_ICA vs. CoCl_2_, and (4) CoCl_2_+EV_ICA vs. CoCl_2_+EV. Enrichment analysis was performed using GO and KEGG gene sets with gene set sizes ranging from 10 to 500 and 1,000 permutations under a fixed random seed. Mitochondria-associated pathways were identified by filtering enriched terms containing at least one of the 22 predefined mitochondrial marker genes within their leading-edge subsets. Pathways with a Benjamini–Hochberg adjusted p-value <0.05 were considered significant and visualized using ggplot2.

#### 2.12.3 Protein-protein interaction (PPI) network analysis

Protein-protein interactions among the 22 mitochondria-associated genes were analyzed via the STRING database (v11.5), integrating multiple evidence channels including experimental data, co-expression, and curated databases. A minimum interaction confidence score of 0.4 was applied to ensure interaction reliability. The resulting network was imported into Cytoscape (v3.10.4) for visualization and topological analysis. Functional clustering was performed to identify biologically coherent gene modules, and hub genes were characterized based on degree and betweenness centrality.

### 2.13 Quantitative real-time polymerase chain reaction

Using TRIzol reagent (Ambion, Carlsbad, CA, USA), we isolated RNA from R28 cells. According to the manufacturer’s protocol, we synthesized cDNA using 500 ng of RNA. Gene expression was quantified using amfiSure qGreen Q-PCR Master Mix (GenDEPOT, Katy, TX, USA) and calculated by the delta CT method. Real-time PCR reactions were performed using a QuantStudio™ Real-Time PCR Instrument (Applied Biosystems, Waltham, MA, USA). The primer sequences used in this study are presented in Supplementary Table 2.

### 2.14 Flow cytometry

Cells were stained with MitoSOX™ Red mitochondrial superoxide indicator (Thermo Fisher Scientific, Waltham, MA, USA) according to the manufacturer’s instructions. Briefly, cells were incubated with 5 μM MitoSOX Red reagent at 37 °C for 10 min in the dark, washed twice with PBS, and resuspended in FACS buffer. Fluorescence intensity was analyzed using a Beckman Coulter flow cytometer, and data were processed using FlowJo software (v11.1.1). A minimum of 10,000 events per sample were acquired. MitoSOX-positive cells were quantified based on fluorescence intensity compared with unstained controls.

### 2.15 Determination of ATP levels

ATP levels were detected by the ATP determination kit (Thermo Fisher Scientific). The production levels were determined by lumenometer (Spectramax iD5; Molecular Devices, San Jose, CA, USA) according to the manufacturer’s instructions.

### 2.16 Statistical analysis

All results are presented as the mean ± standard error of the mean (SEM). Data were analyzed using GraphPad Prism 9 (GraphPad Software, La Jolla, CA, USA). Statistical analyses were performed using GraphPad Prism. Comparisons between two groups were analyzed using the Mann–Whitney U test. Comparisons among multiple groups were analyzed using one-way ANOVA followed by Dunnett’s multiple comparisons test. A p value <0.05 was considered statistically significant.

## 3 Results

### 3.1 Characterization of naïve EV and EV_ICA

To confirm the successful isolation and compare the characteristics of EVs derived from naïve and icariin-loaded EVs, we analyzed their protein markers, size distribution, and surface charge. Western blot analysis demonstrated that both Naïve EV and EV_ICA positively expressed the representative surface markers CD9, CD81, and CD63, as well as the cytosolic marker TSG101 (Figure 1A). This confirms that the isolated particles possess the biochemical properties of EVs. Nanoparticle Tracking Analysis (NTA) revealed that the size distribution of EV_ICA was largely consistent with that of Naïve EV, with both populations showing a peak diameter between 100 and 200 nm (Figure 1B). This indicates that the incorporation of icariin or the treatment process did not significantly alter the physical dimensions of the secreted vesicles. However, a distinct difference was observed in the surface charge of the vesicles. While Naïve EV exhibited a typical strongly negative zeta potential (approximately −25 mV), the zeta potential of EV_ICA was significantly increased to a less negative value (Figure 1C). These results suggest that icariin loading into EVs may induce modifications in the surface molecular composition or charge density of the vesicles.

**FIGURE 1:**
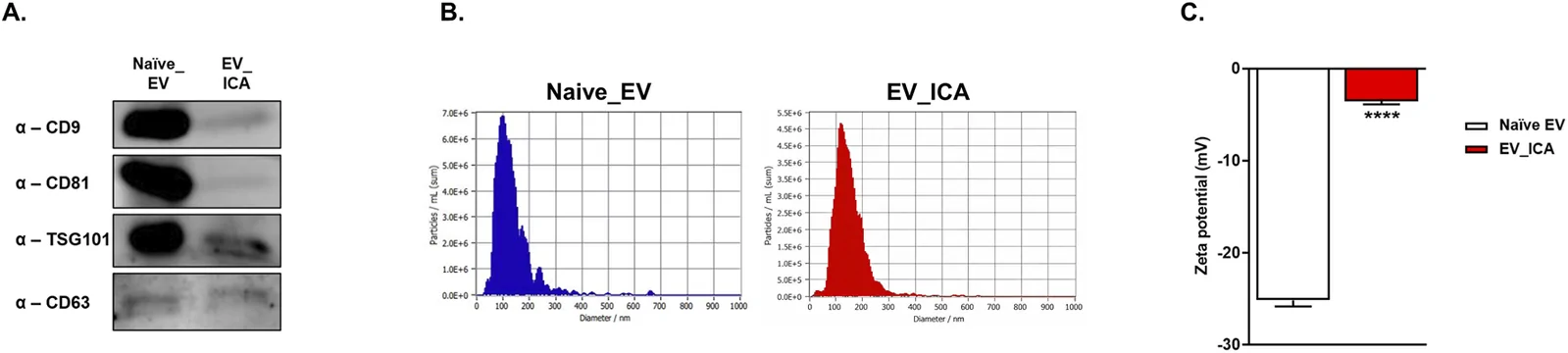
Characterization of EVs. **(A)** Western blot analysis of EVs surface markers (CD9, CD81, CD63) and an internal marker (TSG101). **(B)** Size distributions of Naïve and Icariin-loaded EV based on Nano particle Tracking Analysis (NTA). **(C)** Zeta potential measurements indicating the surface charge of the vesicles. EV_ICA showed a significant shift toward a less negative zeta potential compared to Naïve EV (****p < 0.0001).

### 3.2 EV_ICA attenuates hypoxic injury and restores neuronal and neurotrophic marker expression in R28 cells

To evaluate the neuroprotective potential of EV_ICA, we established an *in vitro* hypoxic injury model using R28 cells treated with CoCl_2_ (200 μM). Western blot analysis revealed that CoCl_2_ exposure significantly downregulated the expression of various neuronal and survival markers, including NeuN, Vegf, Rbpms, Brn-3a, and Bdnf to 0.42-, 0.82-, 0.76-, 0.91-, and 0.66-fold of the control levels, respectively (Figure 2A). Treatment with EV_ICA significantly recovered the protein expression of these markers to 0.85-, 2.47-, 1.66-, 2.29-, and 0.93-fold, respectively, compared to the CoCl_2_ group. In addition, ICA treatment alone further enhanced the expression of several markers, including Vegf (3.04-fold), Rbpms (2.03-fold), Brn-3a (2.98-fold), and Bdnf (1.00-fold). Particularly, EV_ICA treatment showed enhanced recovery than naïve EV treatment for NeuN (0.85 vs. 0.64-fold), Vegf (2.47 vs. 1.59-fold), Rbpms (1.66 vs. 1.17-fold), Brn-3a (2.29 vs. 1.44-fold), and Bdnf (0.93 vs. 0.81-fold), suggesting that icariin-loaded EVs enhance regenerative and neuroprotective efficacy (Figure 2A). The protective effect was further confirmed via immunofluorescence staining (Figure 2B). CoCl_2_ treatment led to a significant increase in Hif-1α intensity, a hallmark of the cellular response to hypoxia. This increase was effectively suppressed in the EV_ICA and ICA-treated groups. Furthermore, the expression of STX12, which was nearly abolished by CoCl_2_ treatment, was restored in cells treated with EV_ICA. Quantitative analysis of the fluorescence intensity corroborated these findings, showing that EV_ICA significantly reduced Hif-1α expression while maximizing Stx12 recovery (Figure 2B). Taken together, these results demonstrate that EV_ICA effectively mitigate hypoxic damage in retinal progenitor cells by modulating Hif-1α and restoring key neurotrophic and regenerative proteins, particularly Stx12, which is consistent with the goal of developing engineered smart EVs for neural tissue regeneration.

**FIGURE 2:**
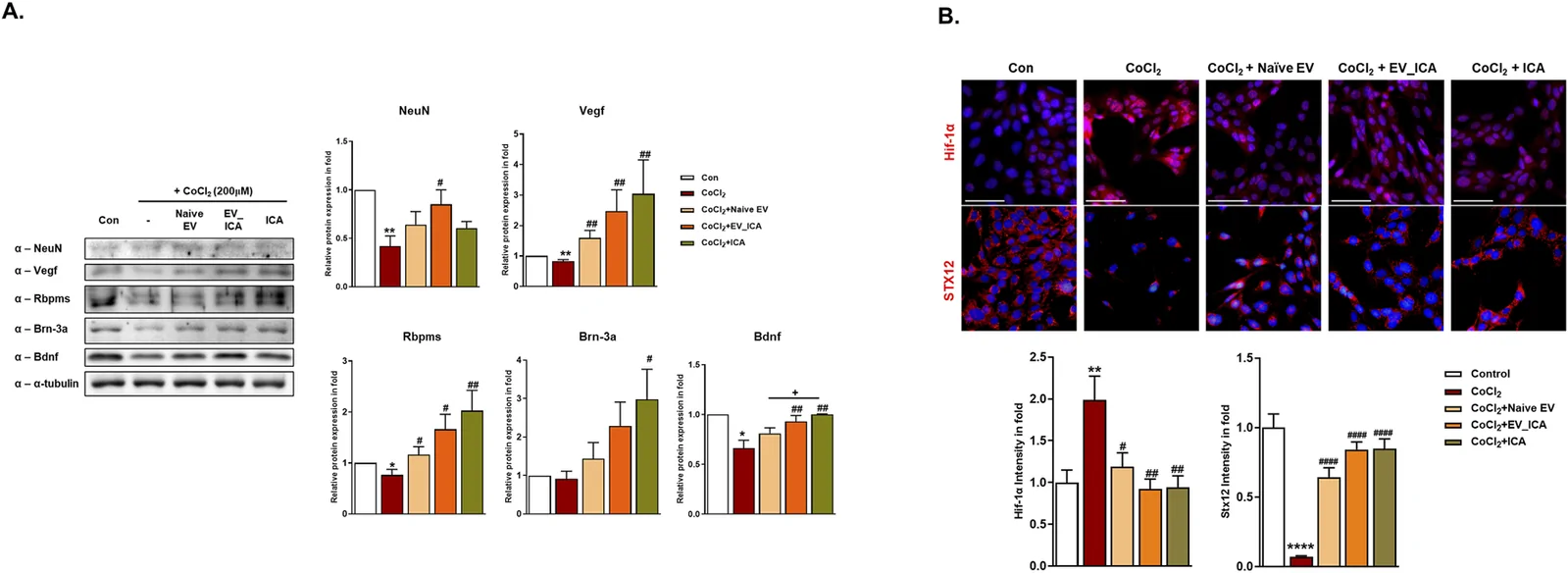
Neuroprotective effects of EV_ICA against CoCl_2_-induced hypoxic injury in R28 cells. **(A)** Neuroprotective and regenerative markers (Stx12, NeuN, Vegf, Rbpms, Brn-3a, Bdnf) levels were presented from R28 lysates with CoCl_2_. Naïve or Icariin-loaded EV were treated in CoCl_2_-exposed R28 cells. After 24 h, a western blot analysis was performed. **(B)** Immunofluorescence staining of Hif-1α (red) and STX12 (red) with DAPI counterstain (blue) (Scale bar = 50 μm). All experiments were independently repeated at least three times, with each experiment performed in technical triplicate. Data are presented as the means ± SEM from three independent biological replicates. Statistical significance was determined using One-way ANOVA (*p < 0.05, **p < 0.01, ****p < 0.001 vs. the control; #p < 0.05, ##p < 0.01, ####p < 0.0001 vs. CoCl_2_; + p < 0.05 vs. Naïve EV).

### 3.3 Development and maintenance of retinal layers in hESC-derived retinal organoids

Based on previous research (Yamasaki et al., 2022), retinal organoids derived from human embryonic stem cells (hESCs) with a structure similar to the human retinal cell layers were developed prior to hypoxia modeling. To enhance the uniform formation of embryonic bodies (EBs), the cells were seeded in a 96-well V-bottom plate and collected via centrifugation from the plate bottom. Currently, it remains challenging for retinal organoids to maintain their retinal layer structure long-term without changes to stratification. To address this issue, we minimized off-target effects as much as possible and excluded organoids that failed to differentiate prior to the experiment to prevent contamination from secreted factors. (Figure 3A). Human embryonic stem cells were confirmed to form stable colonies. These hESCs were dissociated into single cells to form EBs. These embryonic bodies developed into neural structures upon receiving BMP4 signaling. Under the influence of retinoic acid and taurine, retinal organoids were formed (Figure 3B). The organoid clearly distinguishes the directly induced retinal pigment epithelium (RPE) from the retinal layer. The retinal layer, where the retinal layer appears, matures and stably forms its structure within the organoid. Protein-level analyses demonstrated robust expression of BRN3a, PAX6, RBPMS, TUJ1, MAP2, OTX2, and Ki67 in the generated retinal organoids (Figure 3C), confirming their retinal identity. Through this result, we observed an increase in the expression of mature ganglion cells and the coexistence of early-stage and mature neurons, thereby confirming neuronal maturation. Additionally, an increase in progenitor cells and their potential to differentiate into retinal cells, such as photoreceptors and bipolar cells, was confirmed by the expression of OTX2, Ki67, and PAX6. We demonstrate the production of retinal organoids derived from human ES cells for hypoxia modeling and characterize them using retinal markers, showing they are suitable for experimental use.

**FIGURE 3:**
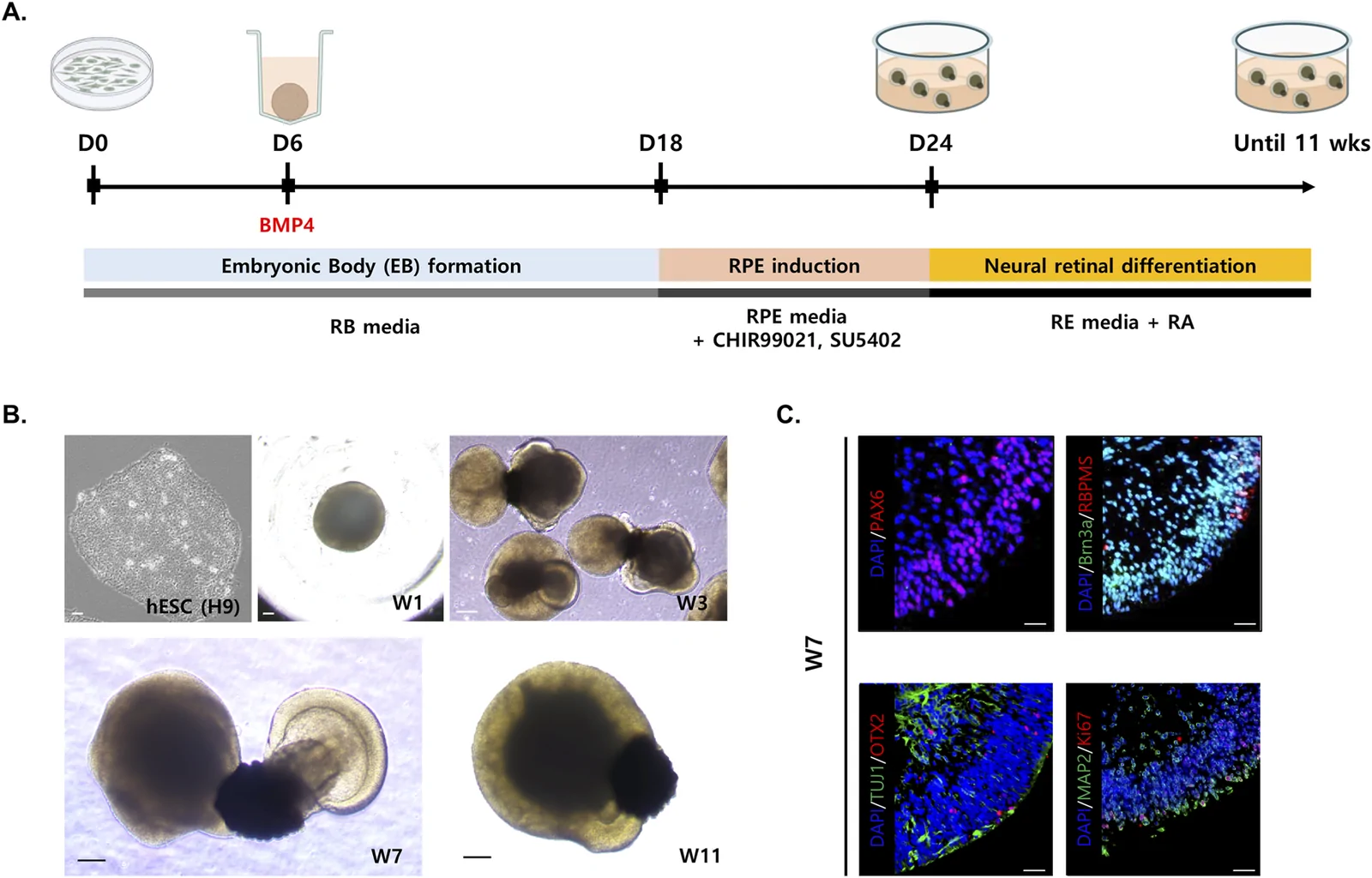
Human embryonic stem cell-derived retinal organoid generation. **(A)** Schematic representation of the steps involved in generating hESC-derived retinal organoids. **(B)** Bright field images of retinal organoids at each week (1,3,7,11 weeks) of maturation from human embryonic stem cells (hESCs). As the organoids matured, there was a significant increase in the formation of retinal ganglion cell (RGC) layers (Scale bar = 10, 20 μm). **(C)** Immunofluorescence analysis of retinal markers at week 7 (W7). Organoids expressed key retinal progenitor and neuronal markers, including PAX6 (red), Brn3a/RBPMS (red), TUJ1/OTX2 (green/red), and MAP2/Ki67 (green/red). Nuclei were counterstained with DAPI (blue) (Scale bar = 50 μm).

### 3.4 CoCl_2_-induced retinal organoid hypoxia modeling

Hypoxia modeling was conducted using retinal organoids 70–100 days, which exhibited high expression levels of BRN3a. Using these retinal organoids, we developed a hypoxia model utilizing CoCl_2_. Organoids were placed in 6 well non-treated culture plates for each group and treated with CoCl_2_ at varying concentrations (0 μM, 600 μM, 1,200 µM) in media for 48 h. At a concentration of 0 μM CoCl_2_, the boundaries of the organoids remained intact over time. However, at concentrations of 600 µM and 1,200 μM, the degree of boundary collapse increased with higher concentrations (Figure 4A). Consistent with this dose-dependent increase in damage, CoCl_2_ induced apoptosis in retinal organoids, beginning in the outermost photoreceptor layer and progressing inward to the progenitor and ganglion cells in the retinal layer, leading to disruption of the retinal layered structure. TUNEL staining confirmed that this structural degradation was accompanied by a significant increase in programmed cell death within the retinal layers by CoCl_2_ treatment (600 μM, 1,200 µM) (Figure 4B). Histological examination revealed collapse of the retinal layers at higher concentrations of CoCl_2_. Immunofluorescence analyses showed a significant decrease in BRN3a expression, a marker of retinal ganglion cells (RGCs), with increasing CoCl_2_ concentrations relative to the 0 µM control. HIF1α expression similarly decreased with increasing concentration and treatment duration. Furthermore, the 48 h group exhibited greater structural disruption and reduced Recoverin and BRN3a expression compared with the 24 h group, regardless of CoCl_2_ concentration (Figure 4C). Therefore, we confirmed that CoCl_2_ treatment of retinal organoids effectively created a hypoxia model, resulting in RGC damage and increased expression of HIF1α. At 48 h after CoCl_2_ treatment, retinal layers were nearly completely disrupted in all groups. Furthermore, BRN3a expression was markedly reduced in the 1,200 µM group even at 24 h, rendering this condition unsuitable for disease modeling and leading to its exclusion from subsequent experiments. Based on these conditions, naïve EV were applied to the hypoxic retina organoid model to confirm the recovery effect. Interestingly, human retinal organoids treated with 200 μg/mL of Naive EVs exhibited a markedly higher fluorescence intensity of the co-localized cyan signal compared to the 100 μg/mL group, suggesting a dose-dependent increase in EV internalization. Concurrently, the number of PAX6-positive cells increased as well. Also, under damage conditions, structural collapse and thinning of the organoid tissue were observed. In the low-dose rescue group, the higher apparent cell density likely reflects pathological nuclear crowding within a contracted laminar area rather than a true increase in cell number. Conversely, treatment with a high concentration of the drug restored structural thickness and neuroepithelial alignment, comparable to the Control group. The relatively lower or more diffuse cell density observed in this group indicates physiological rescue, characterized by healthy cytoplasmic volumes, normal cell-to-cell spacing, and the alleviation of pathological compression. Additionally, this biological interpretation accounts for minor variations arising from the paraffin-sectioning angles of the spherical 3D organoids. These results suggest that naïve EV aid in the recovery of the organoid’s retinal layer by promoting the proliferation and recovery of cells damaged by CoCl_2_ (Figure 4D).

**FIGURE 4:**
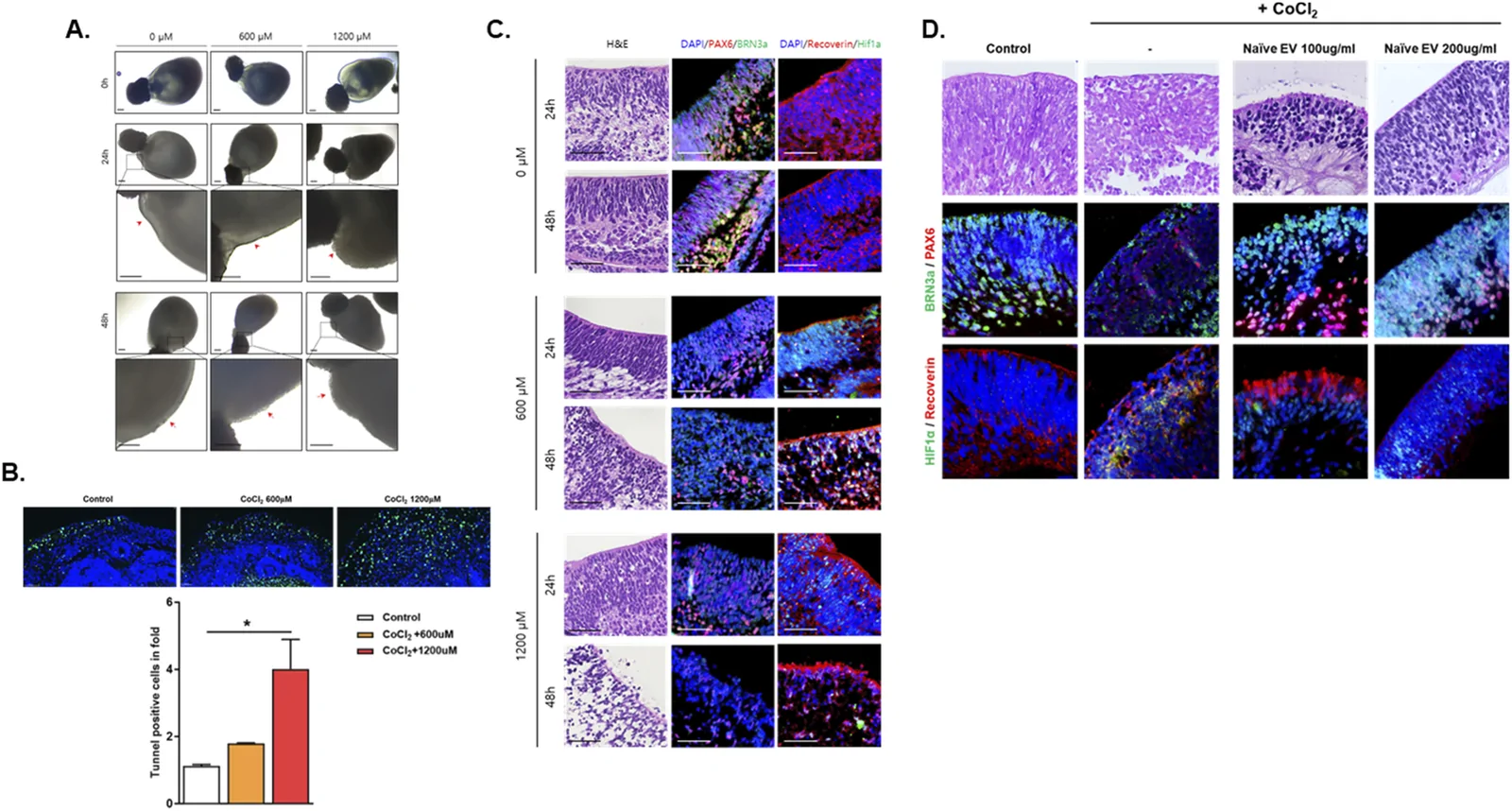
CoCl_2_-induced hypoxia modeling. **(A)** Bright field images of day 70 retinal organoids treated with 0, 600, and 1,200 µM of CoCl_2_ for 0, 24, and 48 h, showing RGC layer collapse over time and with increasing CoCl_2_ concentration. (Scale bar = 10, 20 µm). **(B)** Apoptosis rate in CoCl_2_-induced damaged organoids assessed by TUNEL staining. **(C)** Immunofluorescence analysis of the retinal ganglion cell (RGC) layer in Figure A, including BRN3a, HIF1α, PAX6, and Recoverin for each hypoxia modeling dose test (Scale bar = 10 µm). **(D)** Immunohistochemistry analysis of the retinal ganglion cell (RGC) layer in day 100 retinal organoids, showing BRN3a, HIF1α, Recoverin, and PAX6 expression under CoCl_2_-induced hypoxic conditions with EV or EV_ICA treatment (Scale bar = 10 µm). Representative immunofluorescence images showing the uptake or co-localization of Naive EVs (green) within DAPI-stained nuclei (blue). Note that the co-localization of these markers yields a cyan (light blue) signal, which is more prominently observed in the 200 μg/mL group compared to the 100 μg/mL group.

### 3.5 Transcriptomic profiling reveals functional recovery and signaling modulation by EV_ICA

Based on our initial findings in R28 cells, we further investigated the transcriptional changes in human retinal organoids across four groups: Control, CoCl_2_, CoCl_2_+ Naïve EV, and CoCl_2_+EV_ICA. To elucidate the molecular mechanisms underlying the neuroprotective effects of EV_ICA, we performed a RNA-seq-based transcriptome analysis. Gene Ontology (GO) enrichment analysis of the differentially expressed genes (DEGs) revealed that EV_ICA treatment significantly modulated biological processes essential for retinal integrity (Figure 5A). The most prominently enriched terms were related to sensory perception of light stimulus, visual perception, and photoreceptor cell cilium organization. Chord diagram analysis (Figure 5B) further identified key functional genes such as *RHO* (rhodopsin), *GNAT1*, and *PDE6B*, which are critical for the phototransduction cascade, showing that EV_ICA effectively restore the expression of genes vital for visual function that were suppressed by CoCl_2_-induced hypoxia. KEGG pathway analysis provided deeper insights into the signaling networks activated by EV_ICA. Histograms categorized the regulated transcripts into environmental information processing and organismal systems (Figure 5C). Detailed dot plot analysis (Figure 5D) highlighted the significant enrichment of the Phototransduction pathway and the PI3K-Akt signaling pathway, the latter being a well-known mediator of cell survival and regeneration.

**FIGURE 5:**
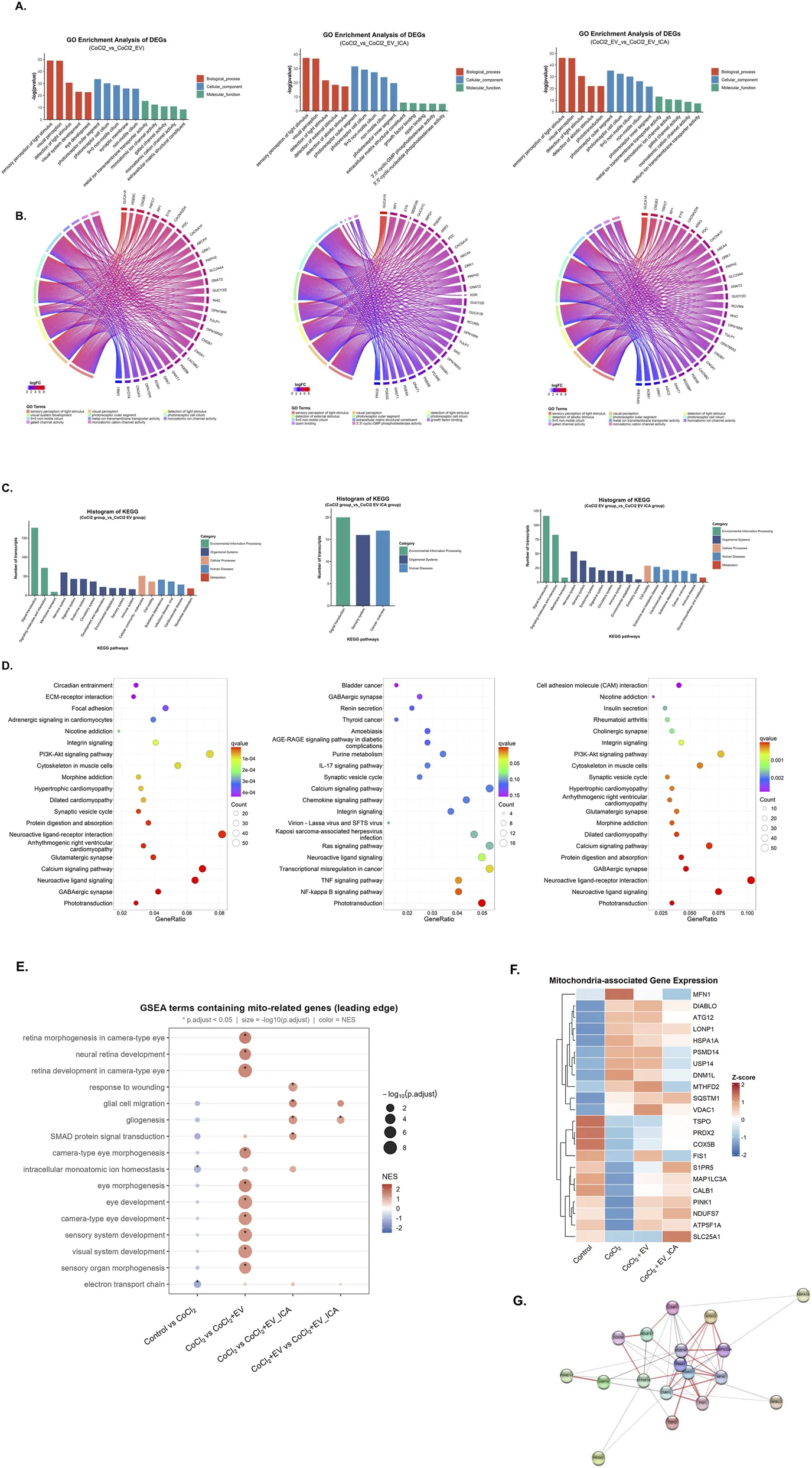
RNA sequencing analysis of hypoxia-damaged day 100 retinal organoids treated with Icariin-loaded EV. **(A)** Gene Ontology (GO) Enrichment Analysis of differentially expressed genes (DEGs) among CoCl_2_, CoCl_2_+Naïve EV, and CoCl_2_+EV_ICA groups. Key enriched terms include sensory perception of light stimulus, visual perception, and photoreceptor cell cilium. **(B)** Chord diagrams illustrate the relationship between enriched GO terms and their associated genes (e.g., *RHO, GNAT1, ABCA4, PDE6B*). **(C)** KEGG pathway histograms showing the distribution of transcripts across functional categories, with significant enrichment in signal transduction and sensory systems. **(D)** KEGG pathway dot plots representing highly significant pathways (q-value <0.05). Notably, EV_ICA treatment significantly modulated pathways related to Phototransduction, PI3K-Akt signaling, and Synaptic vesicle cycle, suggesting a comprehensive rescue of retinal function and survival pathways. **(E)** Gene Set Enrichment Analysis (GSEA) dot plot showing significantly enriched terms containing mitochondria-related genes. While CoCl_2_ treatment suppressed development-related pathways, EV_ICA treatment significantly upregulated terms associated with retina morphogenesis, eye development, and glial cell migration. **(F)** Heatmap of mitochondria-associated gene expression. Z-score visualization reveals that genes involved in mitochondrial dynamics and metabolism were downregulated by CoCl_2_ but effectively restored or modulated by EV_ICA treatment. **(G)** Protein-protein interaction (PPI) network analysis of the differentially expressed mitochondria-related genes, highlighting the functional clusters preserved by EV_ICA.

### 3.6 EV_ICA-mediated mitochondrial rescue under hypoxic retinal injury

To further investigate the mechanism by which EV_ICA rescues human retinal organoids, we focused on mitochondrial integrity and global pathway enrichment using GSEA. GSEA analysis (Figure 5E) revealed that CoCl_2_-induced hypoxia negatively enriched pathways associated with retinal development and organoid maintenance, including retina morphogenesis in camera-type eye, visual system development, and sensory organ morphogenesis. In contrast, EV_ICA treatment significantly restored and positively enriched these developmental gene sets compared with the CoCl_2_ group. In addition, pathways related to response to wounding, glial cell migration, and gliogenesis were enriched following EV_ICA treatment, suggesting activation of tissue repair and neuroprotective responses. The heatmap analysis revealed that CoCl_2_ exposure caused a dramatic decrease in the expression of key mitochondrial genes such as PINK1 (mitophagy), ATP5F1A (ATP synthesis), and MFN1 (mitochondrial fusion) (Figure 5F). EV_ICA treatment significantly restored the expression of these genes toward control levels. Interestingly, genes involved in stress response and proteostasis (e.g., HSPA1A, PSMD14) were also modulated, indicating that EV_ICA help maintain mitochondrial quality control under hypoxic stress. PPI network analysis (Figure 5G) further showed that EV_ICA-restored genes were closely associated with mitochondrial metabolism and survival pathways. These results suggest that EV_ICA protects retinal organoids by preserving mitochondrial function and restoring retinal developmental pathways.

### 3.7 EV_ICA ameliorate mitochondrial dysfunction and oxidative stress induced by hypoxia

We further validated the functional recovery of mitochondria in R28 cells under hypoxic stress. Gene expression analysis via qRT-PCR confirmed that CoCl_2_-induced hypoxia altered the expression of genes involved in mitochondrial proteostasis (HSPA1A, PSMD14), electron transport chain (COX5B, NDUFS7), and dynamics (MFN1, FIS1). Notably, treatment with EV_ICA showed a superior ability to restore these mRNA levels compared to Naïve EV or ICA treatment (Figure 6A). For instance, the expression levels of HSPA1A, MTHFD2 (metabolism), COX5B (respiration), and MFN1 (fusion), which were altered under hypoxic conditions, were substantially restored in the EV_ICA-treated group compared with the ICA-only treatment group, indicating that EV_ICA more effectively recovered mitochondrial structural integrity and respiratory function. Furthermore, we examined the impact of EV_ICA on oxidative stress, a critical mediator of hypoxic damage. MitoSOX staining revealed a marked accumulation of mitochondrial ROS in CoCl_2_-treated cells. Notably, EV_ICA treatment was the only condition that significantly suppressed mitochondrial ROS levels, demonstrating the strongest antioxidative effect among all treatment groups (Figure 6B). In addition, we measured intracellular ATP production. Hypoxia resulted in a significant reduction in ATP content, reflecting severely compromised mitochondrial bioenergetics. However, treatment with naïve EV, ICA, and EV_ICA all restored ATP levels, demonstrating that these engineered vesicles restore the metabolic health of retinal progenitor cells (Figure 6C). Taken together, these results suggest that the neuroprotective mechanism of EV_ICA involves a comprehensive restoration of mitochondrial homeostasis, ranging from gene expression and ROS regulation to the maintenance of cellular energy production.

**FIGURE 6:**
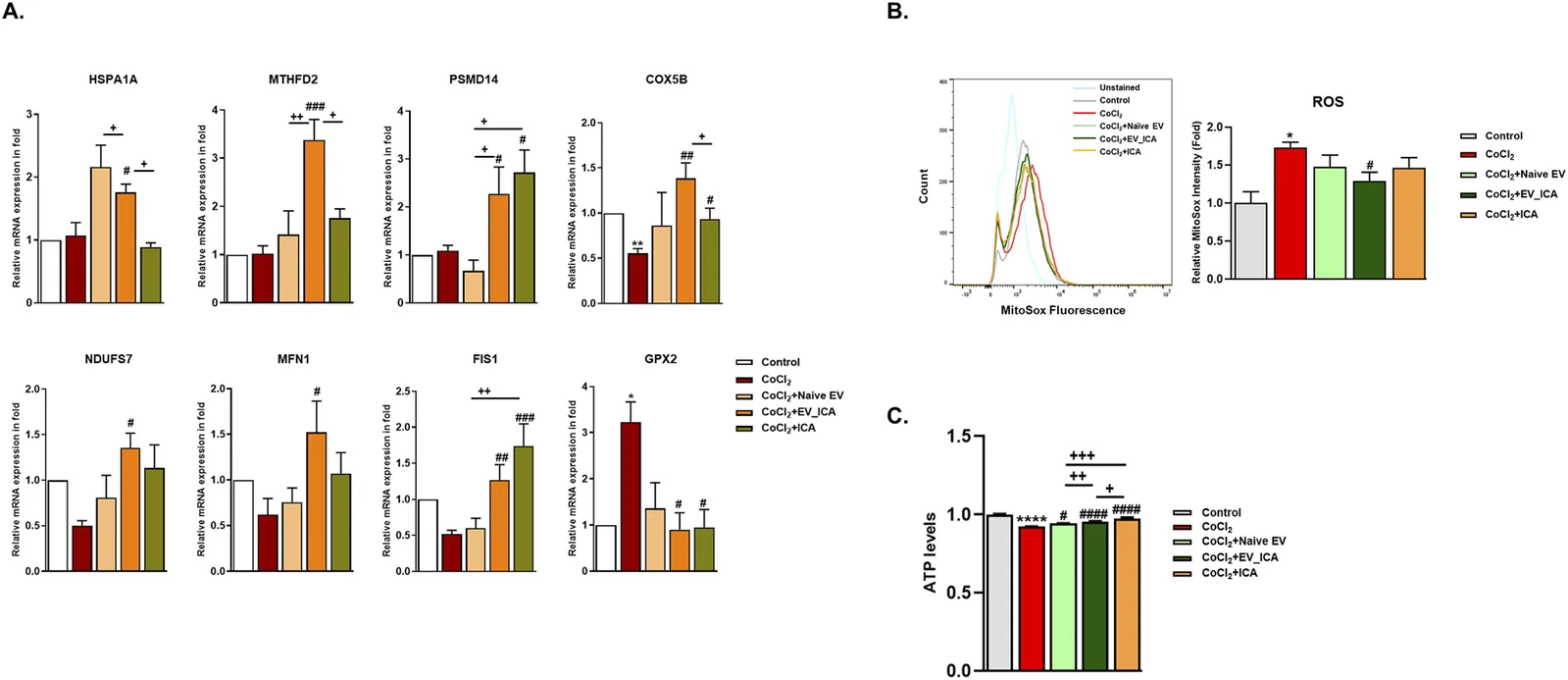
EV_ICA restores mitochondrial function and suppresses oxidative stress under hypoxic conditions. **(A)** Quantification of mitochondria-related genes involved in mitochondrial dynamics, biogenesis, oxidative stress response, and mitophagy across experimental groups. **(B)** Representative flow cytometry plots showing mitochondrial superoxide levels measured by MitoSOX staining in unstained, Control, CoCl_2_, CoCl_2_+ Naïve EV, CoCl_2_ + EV_ICA, and CoCl_2_ + ICA groups. Overlay histogram of MitoSOX fluorescence intensity indicating changes in mitochondrial ROS levels among groups. Quantification ROS levels based on mean fluorescence intensity (MFI). **(C)** Intracellular ATP levels in each experimental group. All experiments were independently repeated at least three times, with each experiment performed in technical triplicate. Data are presented as the means ± SEM from three independent biological replicates. Statistical significance was determined using One-way ANOVA (*p < 0.05, **p < 0.01, ****p < 0.001 vs. the control; #p < 0.05, ##p < 0.01, ###p < 0.005, ####p < 0.001 vs. CoCl_2_; + p < 0.05, ++ p < 0.01, +++ p < 0.005 as indicated).

## 4 Discussion

The therapeutic potential of EVs as drug delivery vehicles has gained significant attention due to their biocompatibility and ability to bypass biological barriers (Alvarez-Erviti et al., 2011; Haney et al., 2015; Wang et al., 2019; Sen et al., 2023). Recent studies have further demonstrated that icariin-loaded EVs can enhance tissue repair and anti-inflammatory responses by improving cargo stability and bioavailability (Dong et al., 2021; Yu et al., 2023). Our previous study demonstrated that surface-modified EVs could promote neuronal recovery in an optic neuropathy model (Park et al., 2026). In R28 cells, c (RGDyK)-modified EVs improved the expression of neuronal markers, including NeuN and RBPMS. Consistent with these findings, EV_ICA also restored NeuN and RBPMS expression under hypoxic conditions in the present study. Although these results were obtained from different studies and cannot be directly compared, they suggest that both surface modification and cargo-loading strategies can enhance the therapeutic efficacy of EVs through distinct engineering approaches. Taken together, EV_ICA preserved retinal neuronal markers, reduced hypoxia-induced cellular stress, and improved mitochondrial function in R28 cells and hESC-derived retinal organoids. To our knowledge, this is the first study to apply MyoCSC-derived EVs for optic neuropathy and to develop icariin-loaded EVs for retinal neuroprotection. These findings suggest that cargo loading is a promising approach to improve the therapeutic effects of EVs for retinal diseases.

The physical characterization of EV_ICA revealed that the loading of icariin did not alter the size distribution or biological features of the vesicles, which remained within the typical range of 100–200 nm and expressed CD9, CD63, CD81 and TSG101. However, compared to Naïve EV, EV_ICA exhibited a significant shift in zeta potential toward a less negative value (approximately −5 mV). Similar alterations in surface charge have been reported in EVs loaded with hydrophobic small molecules such as curcumin, suggesting that incorporation of lipophilic cargo may modify membrane composition or alter the distribution of charged phospholipids on the vesicle surface (Alkhaldi et al., 2024).

A key highlight of our study is the significant restoration of Syntaxin 12 (Stx12) and Bdnf by EV_ICA treatment. Stx12 is a member of the SNARE protein family involved in endosome-to-endosome fusion and intracellular trafficking. Syntaxin proteins have been involved in neuroprotection (Wang et al., 2024; Zhang et al., 2025) and have crucial role in retinal health (Tebbe et al., 2024). Compared to Naïve EV, the EV_ICA has more affected on restoring Stx12 suggests that icariin might specifically enhance the vesicle-mediated transport systems that are crucial for repairing damaged neurons. Furthermore, the suppression of Hif-1α accumulation indicates that EV_ICA effectively attenuates the cellular hypoxia response induced by CoCl_2_, preventing the downstream cascade of apoptosis. Although EV_ICA significantly increased NeuN and RBPMS expression, Brn3a showed only a trend toward recovery. NeuN was the only neuronal marker that showed significantly recovery with EV_ICA than with free icariin. NeuN is a pan-neuronal marker and is not exclusively restricted to retinal ganglion cells. Previous studies have shown that NeuN labels both retinal ganglion cells and displaced amacrine cells, whereas RBPMS is a highly selective marker for RGCs (Rodriguez et al., 2014; Nadal-Nicolas et al., 2023). Therefore, the increased NeuN expression observed following EV_ICA treatment likely reflects the preservation of multiple retinal neuronal populations rather than a selective increase in RGCs alone. This finding suggests that EV_ICA exerts a broad neuroprotective effect on retinal neurons under hypoxic conditions.

The use of hESC-derived retinal organoids was particularly meaningful because the organoids were generated at a developmental stage (day 70–100) enriched for retinal ganglion cell (RGC) differentiation, allowing evaluation of neuroprotective effects in retinal environment. At this stage, retinal organoids exhibit organized retinal lamination and robust expression of early RGC and retinal progenitor markers, including BRN3a, PAX6, and NeuN, which are associated with retinal development. Previous studies have shown that retinal organoids between days 70 and 100 contain a distinct ganglion cell layer enriched with BRN3a-positive RGCs and neuronal markers, closely resembling early human retinal development (Fligor et al., 2018; O'Hara-Wright and Gonzalez-Cordero, 2020; Harkin et al., 2024).

In addition, CoCl_2_, a well-established chemical inducer of hypoxia through HIF-1α stabilization, has been widely used to model hypoxic injury in retinal cells and tissues (Kuehn et al., 2017; Munoz-Sanchez and Chanez-Cardenas, 2019). Therefore, before conducting *in vivo* studies, we established a CoCl_2_-induced hypoxic retinal organoid model to evaluate the therapeutic effects of EV_ICA in a three-dimensional retinal system. As expected, CoCl_2_ treatment induced retinal degeneration and loss of neuronal markers, whereas EV treatment restored BRN3a and PAX6 expression, suggesting improved survival of RGCs and retinal progenitor cells under hypoxic conditions. However, this chemical hypoxia model has a clear limitation because it mostly focuses on HIF‐1α pathways and cannot represent the complex pathology of real retinal diseases, which involve inflammation and blood vessel changes. Also, while hESC-derived retinal organoids show a 3D structure, they still lack blood vessels, immune cells (like microglia), the blood-retinal barrier, and mature nerve connections found in living eyes.

Therefore, before moving forward to the next steps of our research, further evaluations in animal models are essential. Future studies should investigate the best injection routes, how long EVs stay in the eye (biodistribution and retention), and dose-related safety. This will help us confirm if EV_ICA can truly protect retinal structure, save RGCs, and restore visual function in *vivo* models.

To further investigate the functional mechanisms underlying retinal protection, we performed RNA-seq followed by GSEA. Transcriptomic profiling identified the phototransduction pathways as major targets regulated by EV treatment under hypoxic stress. Notably, EV_ICA treatment exhibited a more pronounced restorative effect than naïve EV or ICA alone, particularly in genes associated with photoreceptor development, including *GUGA1B, RCVRN, SAG, and PRCD*. Importantly, EV_ICA more effectively reversed the hypoxia-induced downregulation of mitochondrial genes, such as *PRDX2, PINK1,* and *ATP5F1A,* further highlighting its superior role in mitochondrial quality control (MQC) compared with naïve EV or ICA treatment alone. Hypoxia is well-known to trigger mitochondrial dysfunction, leading to a surge in reactive oxygen species (ROS) and a collapse in ATP production. Our results confirmed that EV_ICA significantly reduced mitochondrial ROS and restored intracellular ATP levels. By promoting mitochondrial fusion (via *MFN1*) and stabilizing the electron transport chain (via *COX5B* and *NDUFS7*), EV_ICA acts as a metabolic stabilizer.

This comprehensive restoration of mitochondrial health appears to be the keystone of the neuroprotective effect observed in our engineered EV system. Previous studies have demonstrated that EVs exert potent anti-inflammatory and mitochondria-protective effects by modulating oxidative stress, promoting mitochondrial homeostasis, and enhancing neurotrophic signaling, including BDNF-associated pathways (Liu et al., 2020; Ahn et al., 2021; Zhang et al., 2021; Sen et al., 2023). In our study, naïve EV exhibited protective effects against hypoxia-induced cellular stress, which were associated with the maintenance of mitochondrial homeostasis-related responses. Additional icariin loading further enhanced the protective efficacy of EVs under hypoxic conditions. Notably, EV_ICA increased the expression of retinal functional and photoreceptor-associated genes, including RCVRN, suggesting that icariin incorporation may contribute to improved retinal cellular responses beyond the effects observed with EV treatment alone. These findings suggest that the intrinsic therapeutic properties of EVs, together with the additional neuroprotective effects of icariin, may contribute to the enhanced therapeutic efficacy of EV_ICA through improved mitochondrial homeostasis and retinal recovery.

## 5 Conclusion

This study demonstrates that EV_ICA exhibits enhanced therapeutic efficacy compared with naïve EVs. The observed therapeutic effects are associated with the restoration of mitochondrial function and neuronal signaling pathways, suggesting that EV_ICA may represent a promising therapeutic strategy for ischemic retinal diseases.

## Data Availability

The RNA-seq data generated in this study have been deposited in the NCBI Gene Expression Omnibus (GEO) under accession number GSE345168.
