# Supplementary material for: Retinal organoid–based evaluation of icariin-loaded extracellular vesicles for hypoxia-induced retinal injury via mitochondrial modulation
Source: Front Cell Dev Biol. 2026 Sep 28;14:1891491. doi: 10.3389/fcell.2026.1891491 (PMC13656086; doi:10.3389/fcell.2026.1891491)
Supplement: Supplementary file 1 [file Table1.docx]

**Table S1. The following tables include all the primary and secondary antibodies use in Immunoblot and Immunofluorescence (IFA) analysis.**

| **Company** | **Primary antibody (Cat.)** | **Host** | **Application** |
| --- | --- | --- | --- |
| Cell Signaling Technology, MA, USA | Anti-CD81 (#56039) | Rabbit | Immunoblot (1:1000) |
|  | Anti-CD9 (#13174) | Rabbit | Immunoblot (1:1000) |
|  | Anti-α-tubulin (#2125) | Rabbit | Immunoblot (1:1000) |
| Genetex, CA, USA | Anti- Vegf (GTX102643) | Rabbit | Immunoblot (1:1000) |
|  | Anti-TSG101 (GTX70255) | mouse | Immunoblot (1:200) |
|  | Anti-Rbpms (GTX118619) | Rabbit | Immunoblot (1:1000) |
|  |  |  | IFA (1:200) |
| Abcam, Cambridge, UK | Anti-Hif-1α (ab179483) | Rabbit | IFA (1:200) |
|  | Anti-Ki67 (ab15580) | Rabbit | IFA (1:200) |
| Invitrogen, MA, USA | Anti-Hif-α (PAI-166601) | Rabbit | IFA (1:100) |
|  | Anti-Pax6 (42-6600) | Rabbit | IFA (1:200) |
|  | Anti-Bdnf (PA5-85730) | Rabbit | Immunoblot (1:1000) |
|  | Anti-Map2 (13-1500) | Mouse | IFA (1:200) |
| Merck KGaA, Darmstadt, Germany | Anti-Brn3a (MAB1585) | Mouse | Immunoblot (1:1000) |
|  |  |  | IFA (1:100) |
|  | Anti-NeuN (MAB377) | Mouse | Immunoblot (1:1000) |
|  | Anti-Recoverin (AB5585-I) | Rabbit | IFA (1:200) |
|  | Anti-Tuj1 (T2200) | Rabbit | IFA (1:200) |
| Proteintech, IL, USA | Anti-Syntaxin12 (14259-1-AP) | Rabbit | Immunoblot (1:1000) |
|  |  |  | IFA (1:100) |
|  | Anti-CD63 (67601-1-IJ) | mouse | Immunoblot (1:1000) |
|  | Anit-Otx2 (13497-1-AP) | Rabbit | IFA (1:200) |
