# Supplementary material for: Retinal organoid–based evaluation of icariin-loaded extracellular vesicles for hypoxia-induced retinal injury via mitochondrial modulation
Source: Front Cell Dev Biol. 2026 Sep 28;14:1891491. doi: 10.3389/fcell.2026.1891491 (PMC13656086; doi:10.3389/fcell.2026.1891491)
Supplement: Supplementary file 2 [file Table2.docx]

**Table S2.** Human primer sequences using quantitative real-time polymerase chain reaction.

| **Genes** | **Primer Sequences** | |
| --- | --- | --- |
| hHSPA1A | Forward  Reverse | 5`- GCGTGATGACTGCCCTGAT-3`  5`- CGGGTTGGTTGTCGGAGTAG-3` |
| hMTHFD2 | Forward  Reverse | 5`- AACCGGCTGCAGACTGACA-3`  5`- CCTTTGGCTGCATTTCCTACA-3` |
| hPSMD14 | Forward  Reverse | 5`- CCTGAACAGCTGGCAATAAAGAAT-3`  5`- AGTACATCCACATGTTCCTCCAAA-3` |
| hCOX5B | Forward  Reverse | 5`- CAATGTACTGGCCCCAAAGG-3`  5`- GGTATTGTCCTCTTCACAGATGCA-3` |
| hNDUFS7 | Forward  Reverse | 5`- TTCGCAAGGTCTACGACCAGAT-3`  5`- CCCTCACCACCGAGTAGGAA-3` |
| hMFN1 | Forward  Reverse | 5`- TTCTACTCCCACTGCTCCTACCA-3`  5`- GTAACGGACGCCAATCCTGTT-3` |
| hFIS1 | Forward  Reverse | 5`- CTGGTGCGGAGCAAGTACAAT-3`  5`- TCCTCCTTGCTCCCTTTGG-3` |
| hGPX2 | Forward  Reverse | 5`- GCAGAACGAGCATCCTGTCTT-3`  5`- AGCTTGGGATCGGTCATGAG-3` |
